# Development, Characterization, and Ex Vivo Assessment of Elastic Liposomes for Enhancing the Buccal Delivery of Insulin

**DOI:** 10.3390/pharmaceutics13040565

**Published:** 2021-04-16

**Authors:** Santosh Bashyal, Jo-Eun Seo, Taekwang Keum, Gyubin Noh, Shrawani Lamichhane, Sangkil Lee

**Affiliations:** 1 College of Pharmacy, Keimyung University, 1095 Dalgubeol-daero, Dalseo-gu, Daegu 42601, Korea; bashyal.santosh18@gmail.com (S.B.); jeseo@kmu.ac.kr (J.-E.S.); gtk02@daum.net (T.K.); rhgyubin@naver.com (G.N.); phr.shrawani@gmail.com (S.L.); 2 Center for Forensic Pharmaceutical Sciences, Keimyung University, 1095 Dalgubeol-daero, Dalseo-gu, Daegu 42601, Korea

**Keywords:** bile salts, elastic liposomes, porcine buccal tissues, buccal permeability, peptide delivery, buccal delivery

## Abstract

Buccal drug delivery is a suitable alternative to invasive routes of drug administration. The buccal administration of insulin for the management of diabetes has received substantial attention worldwide. The main aim of this study was to develop and characterize elastic liposomes and assess their permeability across porcine buccal tissues. Sodium-cholate-incorporated elastic liposomes (SC-EL) and sodium-glycodeoxycholate-incorporated elastic liposomes (SGDC-EL) were prepared using the thin-film hydration method. The prepared liposomes were characterized and their ex vivo permeability attributes were investigated. The distribution of the SC-EL and SGDC-EL across porcine buccal tissues was evaluated using confocal laser scanning microscopy (CLSM). The SGDC-EL were the most superior nanocarriers since they significantly enhanced the permeation of insulin across porcine buccal tissues, displaying a 4.33-fold increase in the permeability coefficient compared with the insulin solution. Compared with the SC-EL, the SGDC-EL were better at facilitating insulin permeability, with a 3.70-fold increase in the permeability coefficient across porcine buccal tissue. These findings were further corroborated based on bioimaging analysis using CLSM. SGDC-ELs showed the greatest fluorescence intensity in buccal tissues, as evidenced by the greater shift of fluorescence intensity toward the inner buccal tissue over time. The fluorescence intensity ranked as follows: SGDC-EL > SC-EL > FITC–insulin solution. Conclusively, this study highlighted the potential nanocarriers for enhancing the buccal permeability of insulin.

## 1. Introduction

During the past decade, the buccal administration of proteins/peptides has gained substantial interest. Compared with the oral route, some advantages of the buccal route of drug administration include better drug delivery, owing to the presence of a dense network of blood vessels that directly drain into the systemic circulation, low susceptibility of drugs to enzymatic degradation, and bypassing the hepatic first-pass effect [1,2]. In addition, the buccal route provides a rapid onset of action and versatility in designing dosage forms. Moreover, the mucosal membranes lining the oral cavity possess robustness and a rapid cell turnover following local stress or damage. In addition, the therapy can be readily terminated when required [3]. The permeability of the buccal mucosa is greater compared with that of the skin. Furthermore, the buccal mucosa has a larger surface area and is easily accessible compared with the nasal, rectal, and vaginal mucosas [4]. Thus, there is a surge in designing noninvasive peptide drug delivery systems, particularly those for buccal administration.

Several options have been explored to enhance the permeability, pharmacokinetics, and targeted selectivity across the buccal membrane. Accordingly, permeation enhancers, adhesive polymers, prodrugs, lipid-based nanocarriers, polymer-based nanocarriers, and hybrid nanocarriers have been designed [5,6,7,8,9,10]. Among these, lipid-based nanocarriers, particularly liposomes that are composed of natural or synthetic phospholipids, with or without cholesterol, have shown tremendous potential in increasing the permeability and bioavailability of peptide drugs [11,12,13]. Liposomes have been reported as attractive nanocarrier systems that can deliver drug molecules via various noninvasive routes, including oral, transdermal, nasal, and buccal routes [14,15,16]. Since the phospholipid is the main component of the cellular membrane, liposomes have been gaining popularity due to their biocompatibility, biodegradability, low toxicity, as well as amphiphilic attributes that allow for the encapsulation of both lipophilic and hydrophilic drugs [17,18]. However, classic liposomes exhibit little or no efficacy when used for protein/peptide delivery, as they just remain confined to the upper layer of the buccal membrane. Therefore, specific nanocarrier vesicles, surface-modified liposomes, or ultra-deformable liposomes are required to enhance the permeability of peptide drugs across alternative routes [19,20]. Recently, the use of ultra-deformable liposomes has gained popularity, owing to the special characteristics of the carrier system, that is, being able to squeeze and penetrate through the membrane barrier prior to releasing the drug into the systemic circulation [21]. These liposomes are also referred to as flexible vesicles or elastic liposomes. Owing to the presence of edge activators, such as Tween 80, Tween 20, Span 80, Span 20, and bile salts, these elastic liposomes can undergo deformation and reformation during penetration across biomembranes [22,23]. In addition, bile salts are bioinspired and biomimetic edge activators, that is, they can destabilize the lipid bilayers of the vesicular system [21,24]. Recently, several approaches have been studied using bile salts to facilitate the transportation of peptides across the buccal membrane [5,8]. The use of insulin–phospholipid complex (IPC)-loaded deformable nanovesicles containing sodium deoxycholate (SDC) resulted in a hypoglycemic effect of up to 4 h in rabbits. As expected, the IPC followed the transcellular route, whereas the deformable nanovesicle followed the paracellular pathway, due to the presence of SDC as an edge activator. The relative pharmacological availability of this preparation in rabbits was 15.53%, indicating the potential of deformable vesicles in maintaining a sustained hypoglycemic effect [25]. Furthermore, previous studies in rabbits revealed the drug-delivery potential of deformable vesicles containing SDC. In these studies, the vesicles exhibited insulin pharmacological availability and relative bioavailability of 15.59% and 19.78%, respectively, which were higher than the corresponding values obtained when using conventional insulin vesicles [26]. Based on these discoveries, we speculated that the use of elastic liposomes could be a suitable approach to increase the buccal permeability of peptide drugs.

In an attempt to innovate more effective buccal delivery nanovesicles, our research group previously developed and explored the potential applications of various elastic bilosomes that were modified with different derivatives of cholic acid, including sodium taurocholate, sodium glycocholate, sodium cholate (SC), sodium glycodeoxycholate (SGDC), or sodium taurodeoxycholate. Among these, the SGDC-modified elastic bilosomes were found to be the most superior at permeating insulin across TR146 buccal cell layers compared with an insulin solution, with a 5.24-fold increase in the apparent permeability [27]. Based on this potential delivery platform, the development and assessment of elastic liposomes incorporating different bile salts (SGDC or SC) are reported herein. The different physical and chemical attributes of dihydroxy bile salts, such as SGDC, and trihydroxy bile salts, such as SC, are summarized in Table 1. Soy lecithin is a natural phospholipid, which is generally regarded as safe for administration and acts as an enhancer for buccal mucosa absorption [28]. Thus, we selected soy lecithin as the phospholipid component to construct the nanoliposomal systems.

The main aim of this study was to develop and characterize SC-incorporated elastic liposomes (SC-EL) and SGDC-incorporated elastic liposomes (SGDC-EL) and evaluate their permeability profiles in porcine buccal tissues. Furthermore, fluorescence-labeled elastic liposomes were also developed and their distribution was assessed using confocal laser scanning microscopy (CLSM) to observe the localization of insulin across the porcine buccal tissues at different time intervals.

## 2. Materials and Methods

### 2.1. Materials

Recombinant human insulin, fluorescein isothiocyanate-labeled insulin (FITC–insulin), and SGDC were purchased from Sigma-Aldrich (St. Louis, MO, USA). Solec 2F-UB (soy lecithin) was obtained from ES Food Ingredients (Gunpo, Korea), and Alfa Aesar SC hydrate was purchased from Thermo Fisher Scientific Inc. (Waltham, MA, USA). All other chemicals were of reagent grade and used without further purification.

### 2.2. Preparation of Elastic Liposomes

Elastic liposomes were prepared using our previously well-established method [27]. Briefly, soy lecithin and a bile salt edge activator, SC or SGDC, (85:15, %*w/w*) were dissolved in chloroform and methanol (3:1) in a round-bottomed flask. Subsequently, the solvent was evaporated using a rotatory evaporator (N-1200BS; EYELA USA, Bohemia, NY, USA) to form a dry lipid film and the remaining traces of solvent were removed under nitrogen gas. The dry lipid film was then hydrated with insulin solution (1.82 mg/mL) at 35 °C in a water bath for 30 min. Finally, the prepared liposomes were extruded five times with an extruder (Avanti Polar Lipids, Inc., Alabaster, AL, USA) using 200 nm polycarbonate membrane filters to fabricate nanosized unilamellar elastic liposomes. Fluorescence-labeled elastic liposomes for the bioimaging analyses were similarly constructed.

### 2.3. Particle Size, Polydispersity Index, and Zeta Potential

Dynamic light scattering (DLS) using a NanoBrook ZetaPALS (Brookhaven Instruments Corp., Holtsville, NY, USA) was used to evaluate the particle sizes, polydispersity indexes (PDIs), and zeta potentials of the synthesized SC-EL and SGDC-EL. Suitable dilutions of the elastic liposomes in distilled water were made prior to each analysis. All studies were performed in triplicate at 25 °C.

### 2.4. Determination of the Entrapment Efficiency and Loading Capacity

The entrapment efficiency (*EE*) and loading capacity (*LC*) of the elastic liposomes were determined. Briefly, the free and total amount of insulin that was initially added to the elastic liposomes was determined by centrifuging the sample dispersions at 200,000× *g* for 2 h at 4 °C (Beckman Optima LE-80K Ultracentrifuge, Beckman Coulter, Inc., Carlsbad, CA, USA). Subsequently, the collected supernatant was analyzed using the human insulin ELISA kit (Mercordia, Uppsala, Sweden) in accordance with the manufacturer’s instructions. The *EE* and *LC* values were calculated using Equations (1) and (2):

(1) $$EE\;\left(\%\right)=\frac{W_{Initial}-W_{Supernatant}}{W_{Initial}}\times 100$$

(2) $$LC\;\left(\%\right)=\frac{W_{Initial}-W_{Supernatant}}{W_{EL}}\times 100$$

where *W_Initial_* indicates the amount of insulin initially added, *W_Supernatant_* refers to the amount of insulin in the supernatant, and *W_EL_* refers to the weight of the elastic liposomes added initially.

### 2.5. Morphological Characterizations

The morphology of the liposomes was determined using transmission electron microscopy (TEM). Briefly, the samples were prestained with 2% (*w/v*) phosphotungstic acid and placed on a carbon-coated Formvar 200-mesh nickel grid. The samples were observed using an H7600 transmission electron microscope (Hitachi, Tokyo, Japan).

### 2.6. Determination of the Deformability Index

The deformability indexes of the SC-EL and SGDC-EL were measured using a single syringe infusion pump (KDS 100 series; KD Scientific Inc., Holliston, MA, USA) according to the previous report [27]. Briefly, both ends of the syringe pumps were fixed such that they could overcome the pressure generated by the extruder. Samples were extruded at a constant flow rate (15.55 mL/h) via a 50 nm polycarbonate membrane filter, and their vesicular sizes were measured using DLS. Finally, the deformability index was calculated using Equation (3):

(3) $$\mathrm{Deformability}\;\mathrm{index}=J\times {\left(\frac{r_{v}}{r_{p}}\right)}^{2}$$

where, *J*, *r_v_*, and *r_p_* are the amount of extruded liposome during 2 min, vesicular size after the extrusion, and the pore diameter of the polycarbonate membrane filter, respectively.

To prepare the control liposomes, we first screened 100% soy lecithin or soy lecithin:cholesterol (85:15 *w/w*%). However, it was not successfully fabricated. Based on our previous studies, we preferred the least effective bile salts that were tested across the TR146 buccal cell layers and screened different ratios of STC with soy lecithin to prepare the control liposomes [27]. Thereby, the minimum amount (5%) of STC was used to fabricate the control liposomes (soy lecithin:STC = 95:5 *w/w*%) via the thin-film hydration method, as described earlier. Thus, 5% STC liposomes were used as control liposomes in deformability studies for calculating the relative deformability index.

### 2.7. Stability of the Elastic Liposomes

The storage stability of the insulin-loaded SGDC-EL and SC-EL was evaluated in terms of the changes in particle sizes and zeta potentials. Briefly, the samples were stored at 4 °C and the mean particle sizes and zeta potential were determined at predetermined time intervals (0, 1, 3, 5, and 7 days) using DLS.

### 2.8. Preparation of Porcine Buccal Tissues

The buccal tissues were prepared according to our previously well-established protocol [29]. Briefly, porcine cheeks (buccal tissues) were collected from freshly sacrificed pigs (6-month-old, ≈110 kg) from a slaughterhouse, stored at 4 °C, and transported to the laboratory. Subsequently, the underlying connective tissues and adipose tissues were removed prior to soaking the buccal tissues in isotonic phosphate-buffered saline (PBS) (pH 7.4) at 60 °C for 1 min. The buccal mucosa was then collected and stored at −20 °C until further use.

### 2.9. Ex Vivo Permeability Studies

**Figure 1:**
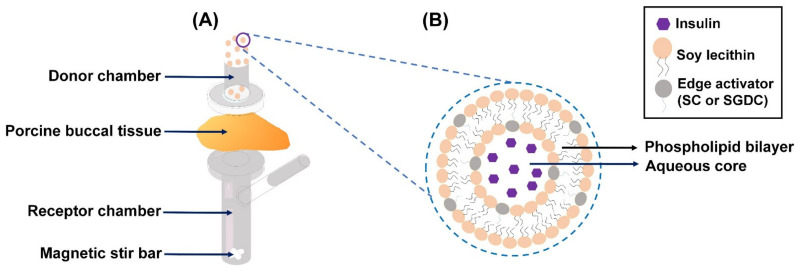
Schematic illustration of the delivery of insulin-loaded elastic liposomes across the porcine buccal tissue: (**A**) Franz diffusion cell system (ex vivo) and (**B**) the elastic liposomes (SC-EL or SGDC-EL).

Ex vivo buccal permeability studies were conducted as illustrated in Figure 1. Permeability assays of the elastic liposomes (SC-EL and SGDC-EL) were conducted across porcine buccal tissues using a vertical static Franz diffusion cell (GlaxoSmithKline, Harlow, UK; 0.79 cm^2^ diffusion area), as described previously [30]. Studies were performed in the direction of the donor to receptor compartment, and the porcine buccal tissue was placed within the donor and receptor compartments of the Franz diffusion cell system, with constant magnetic stirring (600 rpm) at 37 °C. The insulin solution (500 µL), SC-EL, or SGDC-EL (equivalent to 1.82 mg/mL insulin) was added to the donor compartment, whereas the receptor compartment was filled with isotonic PBS (pH 7.4). In addition, to compare the effect of the surfactant in the permeation of insulin, a combination of insulin and a surfactant (insulin + SC or insulin + SGDC; equivalent to 1.82 mg/mL insulin) was also tested across the porcine buccal tissues. Subsequently, the samples were withdrawn at predetermined time intervals (1, 2, 4, 6, and 8 h), and replenished immediately with the same volume of PBS (pH 7.4) to maintain a constant volume in the receptor compartment. A human insulin ELISA kit was used to analyze the collected samples according to the manufacturer’s instructions.

The steady-state flux (*J_s_*) and permeability coefficient (*K_p_*) were obtained using Equations (4) and (5), respectively, where *Q_r_* is the total permeated insulin (ng), *A* is the cross-sectional diffusion area (cm^2^), *t* is the time of exposure (h), and *C_d_* is the initial concentration in the donor chamber (ng·cm^−3^). Additionally, the lag time was calculated from the *x*-intercept of the linear regression line. Furthermore, the *K_p_* value of each formulation was divided with that of the control to obtain the enhancement ratio (ER).

(4) $$J_{s}=\frac{Q_{r}}{A\cdot t}\left(\mathrm{ng}\cdot {\mathrm{cm}}^{-2}\cdot h^{-1}\right)$$

(5) $$K_{p}=\frac{J_{s}}{C_{d}}\left(\mathrm{cm}\cdot h^{-1}\right)$$

### 2.10. Localization Studies in Porcine Buccal Tissues

The distribution of the elastic liposomes across the buccal tissues was analyzed using CLSM. Briefly, the studies were performed using a vertical static Franz diffusion cell system that was set up as described earlier (Figure 1). The donor compartment was loaded with 500 µL of FITC–insulin solution, SC-EL, or SGDC-EL (equivalent to 200 µg/mL FITC–insulin). The experiments were conducted from the donor to the receptor compartment with constant magnetic stirring (600 rpm) at 37 °C. At predetermined time points (1, 4, and 8 h), the buccal tissues were removed, washed with PBS buffer (pH 7.4), and dried with soft paper tissue. Following this, a Tissue-Tek O.C.T. (optimal cutting temperature) compound (Sakura Finetek USA, Inc., Torrance, CA, USA) was introduced to fix the obtained buccal tissues. Subsequently, the buccal tissues were frozen using liquid nitrogen and were cut vertically using a cryostat (Cryotome FE, Thermo Fisher Scientific, Waltham, MA, USA) into 9-µm-thick slices. These sliced portions were laid onto glass slides, fixed with 4% paraformaldehyde, mounted with coverslips, and analyzed using a Zeiss CLSM 800 with Ariyscan (Carl Zeiss Meditec AG, Jena, Germany).

### 2.11. Statistical Analysis

Data are presented as mean ± standard deviation (SD). One-way analysis of variance followed by Tukey’s multiple comparison test was used to determine the statistically significant differences between groups. A *p*-value < 0.05 was considered to be statistically significant.

## 3. Results

### 3.1. Physical Characterization of the Elastic Liposomes

**Figure 2:**
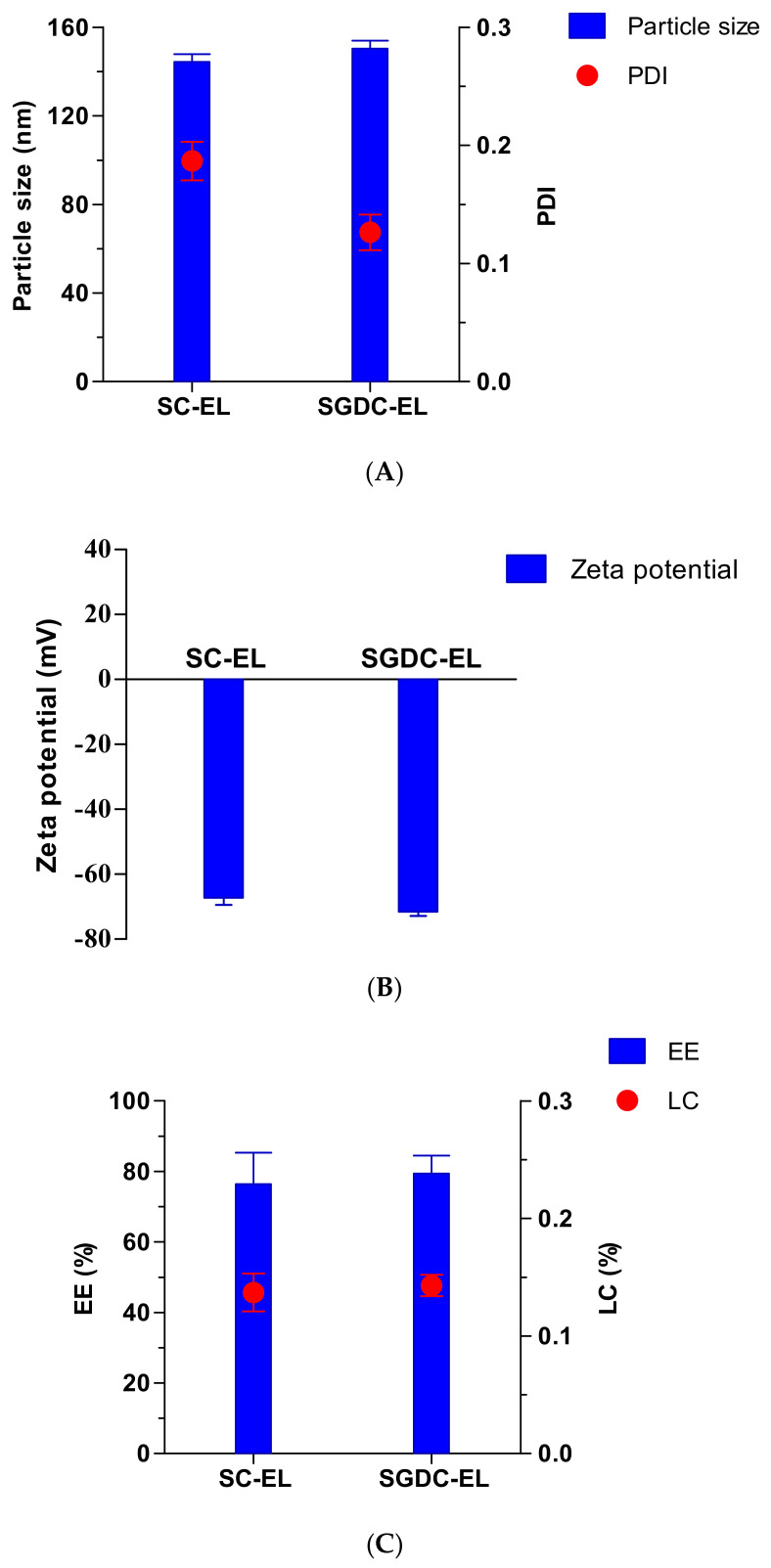
Physical characterizations of the elastic liposomes (SC-EL and SGDC-EL): (**A**) particle size and polydispersity index (PDI), (**B**) zeta potential, and (**C**) entrapment efficiency (*EE*) and loading capacity (*LC*). Data are expressed as mean ± SD (*n* = 3).

Elastic liposomes were prepared using the thin-film hydration method. The physical characterization of the nanocarriers (SC-EL and SGDC-EL) was based on their physical parameters, including their particle sizes, PDIs, EEs, and LCs, as shown in Figure 2. As depicted in Figure 2A, the vesicular sizes of the SC-EL and SGDC-EL were 144.40 ± 3.42 nm and 150.40 ± 3.50 nm, respectively. The PDI of the SC-EL was 0.187 ± 0.016 and that of the SGDC-EL was 0.126 ± 0.015 (Figure 2A). The zeta potential values of the SC-EL and SGDC-EL were −67.37 ± 2.10 mV and −71.64 ± 1.25 mV, respectively (Figure 2B). As presented in Figure 2C, the *EE* and *LC* of the SC-EL and SGDC-EL were 76.45 ± 8.84% and 79.41 ± 5.07%, and 0.137 ± 0.016% and 0.143 ± 0.009%, respectively.

### 3.2. Morphological Characterizations

**Figure 3:**
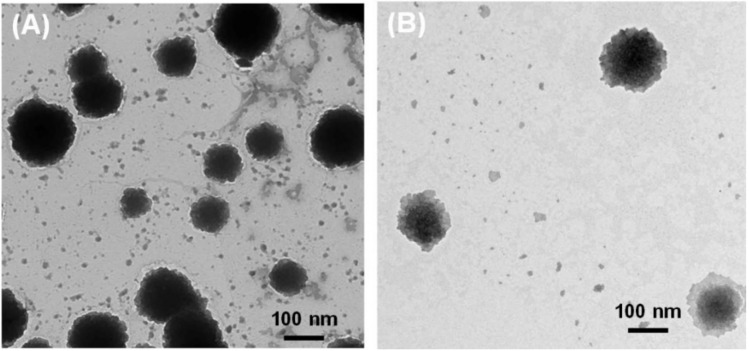
Transmission electron microscopy images of the elastic liposomes: (**A**) SGDC-EL and (**B**) SC-EL.

The morphologies of the elastic liposomes were determined using TEM. As presented in Figure 3, the TEM images revealed SGDC-EL and SC-EL to be spherical and <200 nm in size. However, the SGDC-EL exhibited a more distinct circular appearance in comparison to the SC-EL.

### 3.3. Relative Deformability of Elastic Liposomes

The 5% STC liposomes were characterized based on their particle size and zeta potential. The particle size and zeta potential of the 5% STC liposomes were 150.69 ± 4.68 nm and −57.53 ± 2.09 mV, respectively. The relative deformability indices were determined as compared with the 5% STC liposomes. As listed in Table 2, the deformability index of the SGDC-EL was 1.57- and 1.41-fold greater than the 5% STC liposomes and SC-EL (*** *p* < 0.001 vs. 5% STC liposomes and ^###^ *p* < 0.001 vs. SC-EL), respectively. Thus, the rise in the relative deformability indices was observed in the order of SGDC-EL > SC-EL > 5% STC liposomes.

### 3.4. Stability of the Elastic Liposomes

**Figure 4:**
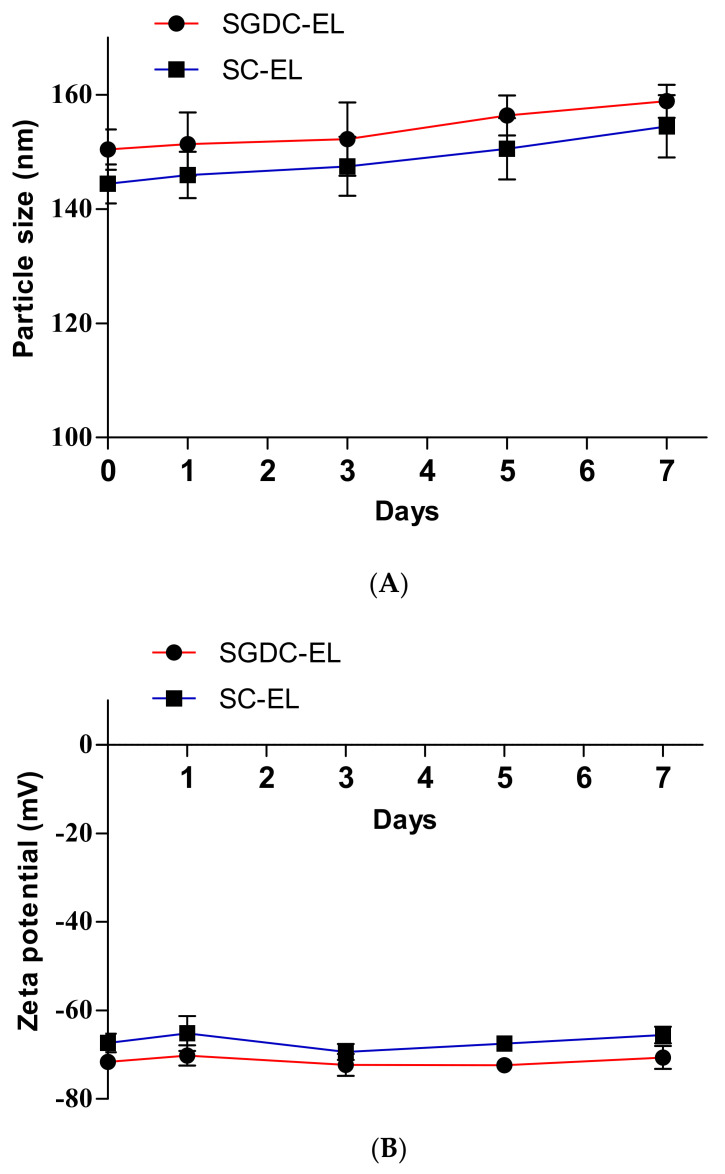
Physical stability of insulin-loaded elastic liposomes (SGDC-EL and SC-EL) at 4 °C. Changes in the (**A**) particle sizes and (**B**) zeta potentials were observed at predetermined intervals over 7 days. Data are expressed as mean ± SD (*n* = 3).

The physical stabilities of the SGDC-EL and SC-EL were evaluated based on changes in the particle sizes and zeta potentials. As shown in Figure 4, the particle sizes and zeta potential of the SGDC-EL and SC-EL were monitored over 7 days. There were no significant changes in the particle sizes or zeta potentials, indicating that these liposomes were physically stable for a week.

### 3.5. Permeability Studies across the Porcine Buccal Tissues

**Figure 5:**
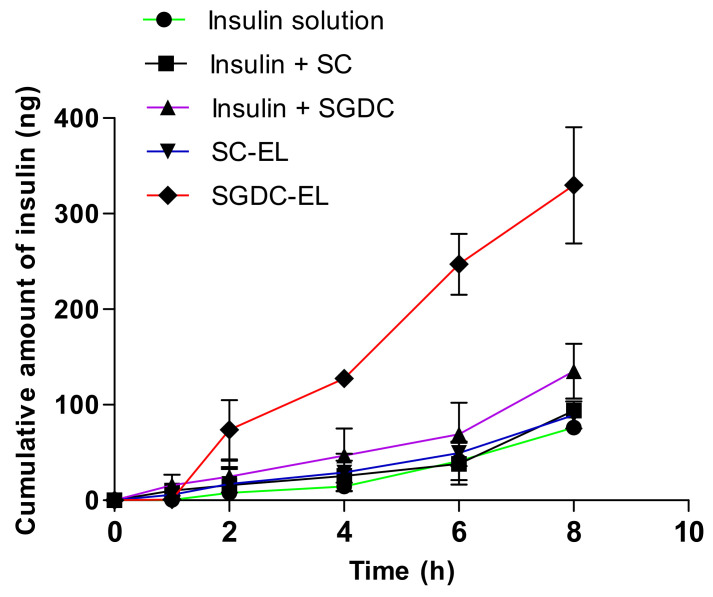
Permeability profiles of insulin-loaded elastic liposomes (SC-EL and SGDC-EL), and the combination of insulin and surfactants (insulin + SC and insulin + SGDC) across the porcine buccal tissues. All studies were performed from the donor to the receptor compartment in PBS buffer (pH 7.4) at 37 °C. Data are presented as mean ± SD (*n* = 3).

The permeability of insulin across the porcine buccal tissues from the elastic liposomes (SC-EL and SGDC-EL) and the combination of insulin and surfactants (insulin + SC and insulin + SGDC) are illustrated as a function of time (Figure 5). The cumulative amount of insulin from SGDC-EL was 4.33-, 3.69-, and 2.44-fold greater in comparison to the amounts from the insulin solution, SC-EL, and insulin + SGDC, respectively. The prospects of the SGDC-EL, SC-EL, insulin + SGDC, and insulin + SC regarding facilitating insulin permeability across the buccal tissues over 8 h was assessed by determining various buccal permeation parameters, such as *J_s_*, *K_p_*, and ER. As listed in Table 3, the rise in these parameters could be ranked as follows: SGDC-EL > insulin + SGDC > insulin + SC > SC-EL > insulin solution. The SGDC-EL exhibited the maximum *J_s_* and was significantly higher than those of the insulin solution and SC-EL (*** *p* < 0.001 vs. insulin solution and ^###^ *p* < 0.001 vs. SC-EL). In addition, the SGDC-EL significantly enhanced the permeability of insulin across the porcine buccal tissues with a 4.33-fold increase in *K_p_* compared with that when using the insulin solution. Similarly, the SGDC-EL significantly enhanced the permeability of insulin across the porcine buccal tissues with a 2.44-fold increase in *K_p_* compared with that of insulin + SGDC (^$$$^ *p* < 0.001 vs. insulin + SGDC). However, there was no significant difference between the SC-EL and insulin + SC in enhancing the permeability of insulin across the porcine buccal tissues. Compared with the insulin solution, the SC-EL also enhanced the permeability of insulin across the porcine buccal tissues, showing a 1.17-fold increase in *K_p_*. The lag time of insulin permeation was reduced to 2.02-, 1.60-, 1.42-, and 2-fold that of the insulin solution, insulin + SC, insulin + SGDC, and SC-EL, respectively.

### 3.6. Localization Studies in the Porcine Buccal Tissues

**Figure 6:**
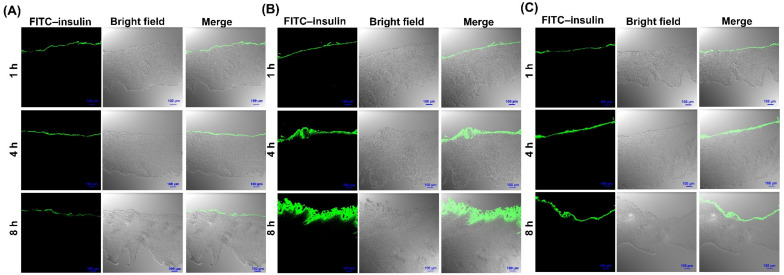
CLSM assessment of the porcine buccal tissues after the application of the (**A**) FITC–insulin solution and FITC–insulin-loaded elastic liposomes ((**B**) SGDC-EL and (**C**) SC-EL) at predetermined time points (1, 4, and 8 h). The scale bar and magnification were 100 µm and ×10, respectively.

The localization of the FITC–insulin in the porcine buccal tissues was evaluated using CLSM. The green fluorescence indicated the localized insulin in the buccal tissues and the studies were performed at predetermined time points (1, 4, and 8 h). As shown in Figure 6, the fluorescence intensity was stronger for the SGDC-EL- and SC-EL-treated groups in comparison to the control group. The fluorescence intensity shifted toward the inner region of the buccal tissues over time in the elastic-liposome-treated groups. However, there were no distinct differences in fluorescence intensity over time in the control group. The images depicted a constant and comparatively weak fluorescence signal on the surface of the epidermis. In addition, the SGDC-EL showed a higher fluorescence intensity compared with the SC-EL, as evidenced by the greater shift in fluorescence toward the inner region of the buccal tissues over time.

## 4. Discussion

Bile salts are generally amphipathic and have been reported as edge activators, that is, they have the potential to undergo deformation and reformation across the intracellular regions of cell membranes [23,31]. Owing to the increase in the fluidity of the membrane, transient pores are formed in the lipid bilayers. Therefore, vesicles that are prepared using hydrophilic surfactants, including bile salts and Tween 80, exhibit higher deformability when compared with those prepared using lipophilic surfactants (Span 80 and Span 85) [32]. Previous studies have reported higher deformability values of vesicles that were developed using phospholipids and edge activators at a ratio of 85:15 %(*w/w*) [32,33]. In addition, our previous studies on insulin-loaded elastic bilosomes revealed these nanocarriers as promising dosage forms for the delivery of insulin across TR146 buccal cell layers. In these studies, SGDC- and SC-incorporated elastic bilosomes enhanced the permeation of insulin by 5.24-fold and 3.2-fold, respectively [27]. Based on the aforementioned findings, the present study involved the fabrication of elastic liposomes (SGDC-EL and SC-EL) and evaluation of their ex vivo permeability characteristics using porcine buccal tissues. As shown in Figure 2, the elastic liposomes were characterized based on their particle sizes, PDIs, zeta potentials, EEs, and LCs. The vesicular sizes of the SC-EL and SGDC-EL were similar and <150 nm, which are ideal for facilitating permeation across biomembranes (Figure 2A) [34]. PDI is a decisive parameter that measures the level of homogeneity. It has been demonstrated that a PDI value greater than 0.3 represents an irregularity in vesicular sizes, whereas PDI values less than 0.1 represent uniformity of vesicular sizes in liposomal systems [35]. The PDI values of the developed nanocarriers were quite close to 0.1, suggesting the presence of uniform and monodispersed systems (Figure 2A). Therefore, the liposomes were of a similar size, had a narrow size distribution, and were homogeneously distributed [36]. As shown in Figure 2B, the zeta potential value of both systems was approximately −70 mV, implying the formation of physically stable elastic liposomes [37]. Due to the presence of anionic bile salts, the liposomal systems were negatively charged. This finding was in agreement with previously published reports [38,39,40,41]. As shown in Figure 2C, the EEs and LCs of the SC-EL and SGDC-EL ranged between 76 and 79%, and 0.137 and 0.143%, respectively, suggesting the successful encapsulation of a high amount of insulin within the hydrophilic core of the formulations. The morphology of the prepared nanocarriers was determined using TEM. As depicted in Figure 3, both formulations exhibited oval or spherical vesicular shapes and had vesicular sizes similar to those determined using DLS. However, the whorls of the SGDC-EL were slightly more distinct than those of the SC-EL.

Deformability is the most important parameter that allows for the internalization of vesicles across the biomembrane [42]. The special ability of elastic liposomes, including deformation and reformation, can be explained via the deformability index [43]. The relative deformability index of elastic liposomes (SC-EL and SGDC-EL) as compared with the 5% STC liposomes are listed in Table 2. The relative deformability index of the SGDC-EL was 1.57 ± 0.05, which was 1.41-fold greater than the SC-EL (^##^^#^ *p* < 0.001 vs. SC-EL). This might have been due to the higher lipophilic nature of dihydroxy bile salt [44]. The stability of liposomes is the major concern for drug delivery scientists. Short-term physical stability studies of SGDC-EL and SC-EL revealed good physical status, i.e., there were no significant changes in the particle sizes and zeta potential over 7 days (Figure 4). In addition, there was no aggregation or sediments while monitoring the physical appearance. However, further studies are necessitated to confirm the long-term stability of these elastic liposomes.

Human buccal tissue is nonkeratinized. Herein, we selected the porcine buccal tissue owing to its morphology, which is consistent with that of the human buccal cheek [29]. Porcine buccal tissue is widely used as an ex vivo model to study the transbuccal permeation of drugs [45,46]. Figure 5 shows the buccal permeability profiles of the insulin-loaded elastic liposomes (SC-EL and SGDC-EL), and the combination of insulin and surfactants (insulin + SC and insulin + SGDC). The SGDC-EL showed the highest permeated amount of insulin compared with the SC-EL and insulin solution. Table 3 summarizes the permeation parameters that were determined from permeability studies. The SGDC-EL exhibited the highest *J_s_*, *K_p_*, and ER values compared with those of other tested samples. The *J_s_* and *K_p_* of the SGDC-EL were 4.33- and 3.69-fold higher than those of the insulin solution and the SC-EL, respectively. Accompanied by the increased *J_s_*, *K_p_*, and ER, the SGDC-EL revealed the shortest lag time (0.48 h), implying the more rapid achievement of a steady state in the buccal tissue permeation than that of the SC-EL and other tested groups. Lastly, the ER values of the SGDC-EL and SC-EL were 4.33 and 1.77, respectively, compared with that of the insulin solution. Additionally, the SGDC-EL showed a greater ER than that of a combination of insulin and surfactant (SGDC). These findings might be attributed to the nature and enhancing effects of the dihydroxy bile salt. As shown in Table 1, the dihydroxy bile salt has fewer H-bond donors and acceptors than the trihydroxy bile salt, leading to an increase in the lipophilicity of the liposomal system, as per Lipinski’s rule of five. Thus, this may be the one reason for the enhancement of the insulin permeability with the use of the SGDC-EL compared with that obtained using the SC-EL. Additionally, the SGDC-EL revealed the greatest relative deformability index compared with the SC-EL and control (Table 2). Thus, the SGDC-EL permeated higher amounts of insulin than that of the SC-EL. This means that the SGDC-EL had a greater ability to squeeze or undergo deformation across the intracellular regions of buccal tissues and delivered intact vesicles across the tissues. Thereby, the permeability profiles of these elastic liposomes were in accordance with the observation of deformability studies. Moreover, another study reported by Yang et al. revealed an underlying mechanism for the internalization of insulin-loaded SDC-containing deformable liposomes across buccal tissues. The transbuccal hydration force and fusion of elastic vesicles within the buccal membrane might be the plausible cause for the enhancement of insulin permeability by deformable liposomes [26]. Similarly, the combination of insulin and surfactants (insulin + SGDC and insulin + SC) also enhanced the permeation of insulin across the porcine buccal tissues, displaying a 1.77- and 1.23-fold increase in the permeability coefficient compared with the insulin solution, respectively, suggesting the enhancing effect of the surfactants. Bile salts act as permeation enhancers and have the potential to increase the absorption of drugs via the extraction of membrane proteins or lipids, membrane fluidization, induction of aqueous channels, and formation of reverse micelles [44]. Furthermore, a previous study on the transbuccal delivery of decitabine revealed the superiority of dihydroxy bile salts (SGDC and SDTC) over trihydroxy bile salts (SGC and STC) by demonstrating a 38-fold increase in the apparent permeability compared with the control. Owing to the postulated mechanisms of decitabine permeation in the presence of bile salts, including the extraction and denaturation of proteins, solubilization and micellar entrapment of intercellular lipids, and swelling of tissues, the increase in the apparent permeability could have been higher [47]. However, herein, compared with a combined effect of insulin and SGDC, the SGDC-EL were better at facilitating insulin permeability, with a 2.44-fold increase in the permeability coefficient across the porcine buccal tissue, implying that the major driving force of enhancement was due to the deformability of elastic liposomes. Overall, the findings confirmed that the enhanced permeability of insulin with SGDC-EL could be attributed to several factors, including the deformability, lipophilic nature, and enhancing effect of the surfactant (SGDC).

The insulin solution (or the smallest insulin molecules) had diffused and permeated to some extent. However, compared with the elastic liposomes, the cumulative amount permeated by the insulin solution was low. This might have been owing to the low ability to cross the physical barrier (buccal mucosa) due to the hydrophilic nature of insulin and without following the specific pathway for permeation across the biomembrane. However, the elastic liposomes had the potential to undergo deformation and reformation across the intracellular regions of buccal mucosa. In addition, in release studies, the insulin solution may reveal greater diffusion than liposomes, or liposomes showed sustained release profiles. In contrast, during permeability studies, lipid-based formulation (liposomes) or other delivery carriers revealed greater permeability profiles due to their specific transport pathway. In this study, we did not measure the insulin release from the elastic liposomes. However, it is a well-known mechanism that the liposomes can easily permeate across the biomembrane via different pathways, i.e., transcellular, paracellular, or by fusion with the lipid membrane. Thus, we assumed that the elastic liposomes had internalized across the porcine buccal tissues and released the insulin at the basolateral compartment via any of these three mechanisms or a combination thereof. In addition, during the permeation study, at different time points (0.5, 1, 2, 4, 6, and 8 h), 500 µL of samples were withdrawn from the basolateral chamber and the amount of permeated insulin across porcine buccal tissues was determined using ELISA. This means that the insulin-loaded elastic liposomes released the insulin at the basolateral compartment. From Figure 5 (ex vivo permeability studies), we observed that the lag time or the time that showed clear differences in the permeation amounts was 0.48–0.97 h. In addition, we observed that the pattern of the cumulative permeated amount or the steady-state flux was increased throughout the study. Therefore, we can speculate that the elastic liposomes were permeated across the porcine buccal tissues continuously and released the insulin at the basolateral compartment. Furthermore, we are designing another study on diabetic rabbit models, which includes investigating the release profiles, long-term stability, thermodynamic stability of these elastic liposomes, and underlying mechanism for increasing the permeability, as well as pharmacodynamic and pharmacokinetic studies with these elastic liposomes.

The distribution of the SGDC-EL and SC-EL across the porcine buccal tissues was determined using CLSM. The localization of insulin in the buccal tissues was determined at predetermined time points (1, 4, and 8 h). As shown in Figure 6, the FITC–insulin solution induced the least fluorescence across the buccal tissues, was not abundant with time, and remained only in the upper layer of the epidermis over 8 h. However, upon encapsulation of the FITC–insulin in SGDC-EL and SC-EL, there was an intense green fluorescence across the buccal tissues, which darkened and shifted into the inner surface of the buccal tissues over time. Interestingly, the greater fluorescence intensity of localized FITC–insulin was observed in buccal tissues treated with the SGDC-EL after 8 h compared with those treated with the SC-EL. Overall, the findings depicted that the SGDC-EL exhibited the highest fluorescence intensity of FITC–insulin across the buccal tissues. The increase in fluorescence intensity was found in the order of SGDC-EL > SC-EL > FITC–insulin solution. These observations were in agreement with the findings from ex vivo permeability studies using porcine buccal tissues.

## 5. Conclusions

Herein, we developed elastic liposomes (SGDC-EL and SC-EL) using the thin-film hydration approach and successfully characterized them based on their particle sizes, PDIs, zeta potentials, EEs, and LCs. In addition, the morphology of the elastic liposomes was evaluated based on TEM. The SGDC-EL were most efficient as a nanocarrier system for the permeation of insulin across porcine buccal tissues. Compared with the SC-EL, the SGDC-EL showed greater potential to enhance the permeability of insulin based on the 3.70-fold increase in the *K_p_* across the porcine buccal tissues. Furthermore, the SGDC-EL also significantly enhanced the permeation of insulin across the porcine buccal tissue, revealing a 2.44-fold increase in permeability coefficient compared with a combination of insulin and surfactant (SGDC), indicating the major driving force of enhancement was owing to the deformability of elastic liposomes. However, further investigations are required to elucidate the underlying mechanism for the transport of insulin across porcine buccal tissues. Overall, our study depicted the potential insight of nanomedicine as a basis for the generation of buccal delivery systems for peptide drugs.

## Figures and Tables

**Table 1:** Comparison of physical and chemical attributes of trihydroxy (SC) and dihydroxy (SGDC) bile salts. Mol wt., molecular weight; CMC, critical micelle concentration.

Attributes	SC Hydrate	SGDC
Mol wt. (g/mol)	448.58	471.61
H-bond donor	4	3
H-bond acceptor	6	5
CMC at 20–25 °C (mM)	9–15	2.1
Chemical structure	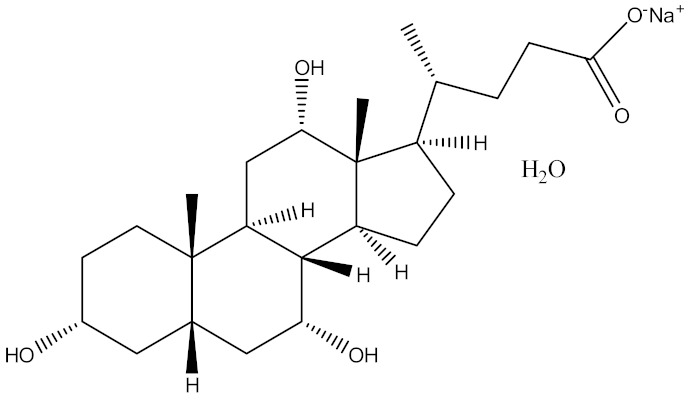	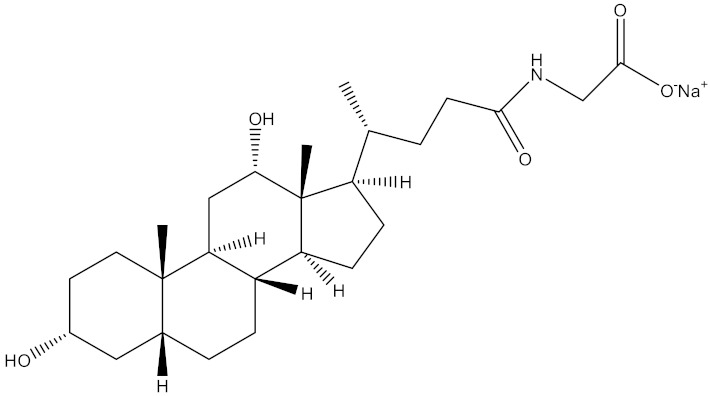

**Table 2:** Relative deformability index of the elastic liposomes (SC-EL and SGDC-EL). Data are expressed as mean ± SD (*n* = 3). * *p* < 0.05 vs. 5% STC liposomes, *** *p* < 0.001 vs. 5% STC liposomes, and ^###^ *p* < 0.001 vs. SC-EL.

Formulation	Relative Deformability Index
5% STC liposomes	1.00 ± 0.04
SC-EL	1.11 ± 0.02 *
SGDC-EL	1.57 ± 0.05 ***^,^^###^

**Table 3:** Permeation parameters that were calculated from the permeability studies of insulin-loaded elastic liposomes (SC-EL and SGDC-EL) and the combination of insulin and surfactants (insulin + SC and insulin + SGDC) across the porcine buccal tissues. Data are expressed as mean ± SD (*n* = 3). *** *p* < 0.001 vs. insulin solution, ^###^ *p* < 0.001 vs. SC-EL, and ^$$$^ *p* < 0.001 vs. insulin + SGDC. *J_s_*, steady-state flux; *K_p_*, permeability coefficient; ER, enhancement ratio.

Formulation	*J_s_* (ng·cm^−2^·h^−1^)	*K_p_* ((cm/h) × 10^−5^)	Lag time (h)	ER
Insulin solution	12.05 ± 0.70	1.32 ± 0.08	0.97 ± 0.14	1.00
Insulin + SC	14.85 ± 1.12	1.63 ± 0.12	0.77 ± 0.39	1.23
Insulin + SGDC	21.36 ± 4.53	2.35 ± 0.50	0.68 ± 0.48	1.77
SC-EL	14.13 ± 2.24	1.55 ± 0.25	0.80 ± 0.24	1.17
SGDC-EL	52.18 ± 9.63 ***^, ###, $$$^	5.73 ± 1.06 ***^, ###, $$$^	0.48 ± 0.24	4.33

## Data Availability

Not applicable.
